# Decreasing the Burden of Type 2 Diabetes in South Africa: The Impact of Taxing Sugar-Sweetened Beverages

**DOI:** 10.1371/journal.pone.0143050

**Published:** 2015-11-17

**Authors:** Mercy Manyema, J. Lennert Veerman, Lumbwe Chola, Aviva Tugendhaft, Demetre Labadarios, Karen Hofman

**Affiliations:** 1 PRICELESS SA- MRC/Wits Rural Public Health and Health Transitions Research Unit (Agincourt), School of Public Health, Faculty of Health Sciences, University of the Witwatersrand, Johannesburg, South Africa; 2 School of Public Health, University of Queensland, Brisbane, QLD, Australia; 3 Population Health, Health Systems and Innovation (PHHSI), Human Sciences Research Council, Capetown, South Africa; Erasmus University Rotterdam, NETHERLANDS

## Abstract

**Introduction:**

Type 2 diabetes poses an increasing public health burden in South Africa (SA) with obesity as the main driver of the epidemic. Consumption of sugar sweetened beverages (SSBs) is linked to weight gain and reducing SSB consumption may significantly impact the prevalence of obesity and related diseases. We estimated the effect of a 20% SSB tax on the burden of diabetes in SA.

**Methods and Findings:**

We constructed a life table-based model in Microsoft Excel (2010). Consumption data from the 2012 SA National Health and Nutrition Examination Survey, previously published own- and cross-price elasticities of SSBs and energy balance equations were used to estimate changes in daily energy intake and its projected impact on BMI arising from increased SSB prices. Diabetes relative risk and prevalent years lived with disability estimates from the Global Burden of Disease Study and modelled disease epidemiology estimates from a previous study were used to estimate the effect of the BMI changes on diabetes burden. Diabetes cost estimates were obtained from the South African Council for Medical Schemes. Over 20 years, a 20% SSB tax could reduce diabetes incident cases by 106 000 in women (95% uncertainty interval (UI) 70 000–142 000) and by 54 000 in men (95% UI: 33 000–80 000); and prevalence in all adults by 4.0% (95% UI: 2.7%-5.3%). Cumulatively over twenty years, approximately 21 000 (95% UI: 14 000–29 000) adult T2DM-related deaths, 374 000 DALYs attributed to T2DM (95% UI: 299 000–463 000) and over ZAR10 billion T2DM healthcare costs (95% UI: ZAR6.8–14.0 billion) equivalent to USD860 million (95% UI: USD570 million–USD1.2 billion) may be averted.

**Conclusion:**

Fiscal policy on SSBs has the potential to mitigate the diabetes epidemic in South Africa and contribute to the National Department of Health goals stated in the National NCD strategic plan.

## Introduction

The prevalence of type 2 diabetes mellitus (T2DM), a chronic disorder of glucose metabolism, is growing [1–3]. In 2005 the World Health Organisation (WHO) estimated that 2% of all deaths worldwide were diabetes-related [4]. Global projections show that diabetes prevalence is set to double from 285 million in 2010 to 592 million in 2035, with the sub-Saharan African (SSA) region bearing the brunt of this increase and South Africa (SA) at the forefront [5,6]. This increase in diabetes rates has been driven largely by economic development and lifestyle changes and has been closely linked to the upsurge in obesity [7,8]. In SA the prevalence of diabetes in adults nearly doubled from 5.5% to 9% between 2000 and 2009 respectively [9,10]. In 2009 over 73 000 disability-adjusted life years (DALYs) were attributable to T2DM and its sequelae, and diabetes-related amputations and cases of blindness were estimated at approximately 2000 and 8000 respectively [10].

Diabetes and other non-communicable diseases (NCDs) pose a significant economic burden. The costs of T2DM are partly direct, such as hospital and medication costs and disability grants incurred by individuals, families or governments, and partly indirect, via work absenteeism, time spent caring for sick relatives, and reduced productivity. About 76% of diabetes deaths in SSA occur in people younger than 60 years old, the most economically active demographic segment of the population [7]. Total health expenditure for diabetes for adults in SSA is projected to increase by approximately 50% between 2010 and 2030 [11]. In SA, these costs are projected to be between 1.1 to 2 billion USD in 2030 [11]. The average hospitalisation costs per patient with diabetes in SA in 2009 were about R27 000 (2 250 USD) compared to R18 000 (1 500 USD) for non-diabetic patients [12].

Research suggests that sugar-sweetened beverage (SSB) consumption is a risk factor for overweight and obesity as well as several cardio-metabolic conditions, especially T2DM [13–16]. Both overweight and obesity are the main drivers of the T2DM epidemic [8]. SSBs contribute substantially to total per capita sugar and energy consumption. Based on manufacturer’s food labels, a 330ml can of carbonated sweetened soft drink contains approximately 40g of sugar and the same size container of sweetened fruit juice close to 45g of sugar. Due to this high content of added sugars, and inadequate compensation for total energy intake at subsequent meals, SSBs are believed to predispose to T2DM firstly through weight gain and increased adiposity, which have been linked to insulin resistance and inflammation [8,13]. Secondly, the rapidly absorbable sugars contained in SSBs may themselves contribute to high dietary glycaemic load leading to inflammation, impaired β-cell function and insulin resistance [13,17]. The Black Women’s Health Study showed that daily consumption of two or more SSBs increased the risk of developing T2DM by 24% compared to consuming fewer than one SSB per month [18]. A meta-analysis of cohort studies has documented a significant positive association between dietary glycaemic index and glycaemic load and the risk of T2DM [19].

The consumption of SSBs has increased worldwide and SA is no exception. In 2002, South Africans consumed 183 Coca-Cola products per person per year, which increased to 260 products in 2012 putting South Africa in the top ten consumers of Coca-Cola products. The worldwide average in 2012 was 94 [20]. These estimates are based on a United States of America (USA) eight fluid ounce serving; approximately 250ml. Euromonitor data also shows that off-trade sales of soft drinks in South Africa totalled 4,206 million litres in 2013, up from 3,620 in 2008 [21]. A 2014 study also showed that total soft drink consumption in SA increased by 69% from 55 litres/capita per year in 1999 to 92.9 litres/capita per year in 2012 [22].The proportion of adults drinking SSBs in rural areas approximately doubled from 25% to 56% in women and from 33% to 63% in men between 2005 and 2010 [23].

Evidence from modelling and meta-analysis studies suggests that fiscal policy, such as taxing SSBs, can reduce consumption and consequently total energy intake with attendant weight loss [24–28]. Studies in the United Kingdom and Ireland have shown the potential of an SSB tax to reduce obesity by 1.3% (20% tax) and 1.3% (10% tax) respectively [24,26]. In India a 20% SSB tax was projected to reduce overweight and obesity by 3% and the incidence of T2DM by 1.6% over the period of 2014–2023 [28]. In SA a modelling study has shown that a 20% SSB tax could potentially reduce obesity by 2.4% in adults but there are currently no estimates of the potential impact on the burden of T2DM [29].

Fiscal and regulatory policy tools have been successfully used in South Africa for improving public health. Increases in tobacco excise tax and tobacco control regulations decreased aggregate cigarette consumption by a third between 1993 and 2003, and per capita consumption by 40%. The instrument with the biggest impact was excise taxation [30]. In 2011 and 2013 regulations were passed to limit trans-fatty acid and salt content in food respectively [31–33]. The National Department of Health (DOH) Strategic Plan for the Prevention and Control of NCDs 2013–17 lists taxes on foods high in sugar as a potential “best buy” for addressing diet and obesity [32]. In line with these approaches, the Minister of Health has recently called for regulations on foods high in sugar [34]. The aim of this study is to model the potential impact of a 20% SSB tax on the burden of type 2 diabetes in South African adults, and on the associated healthcare costs.

## Methods

### Model overview

**Fig 1:**
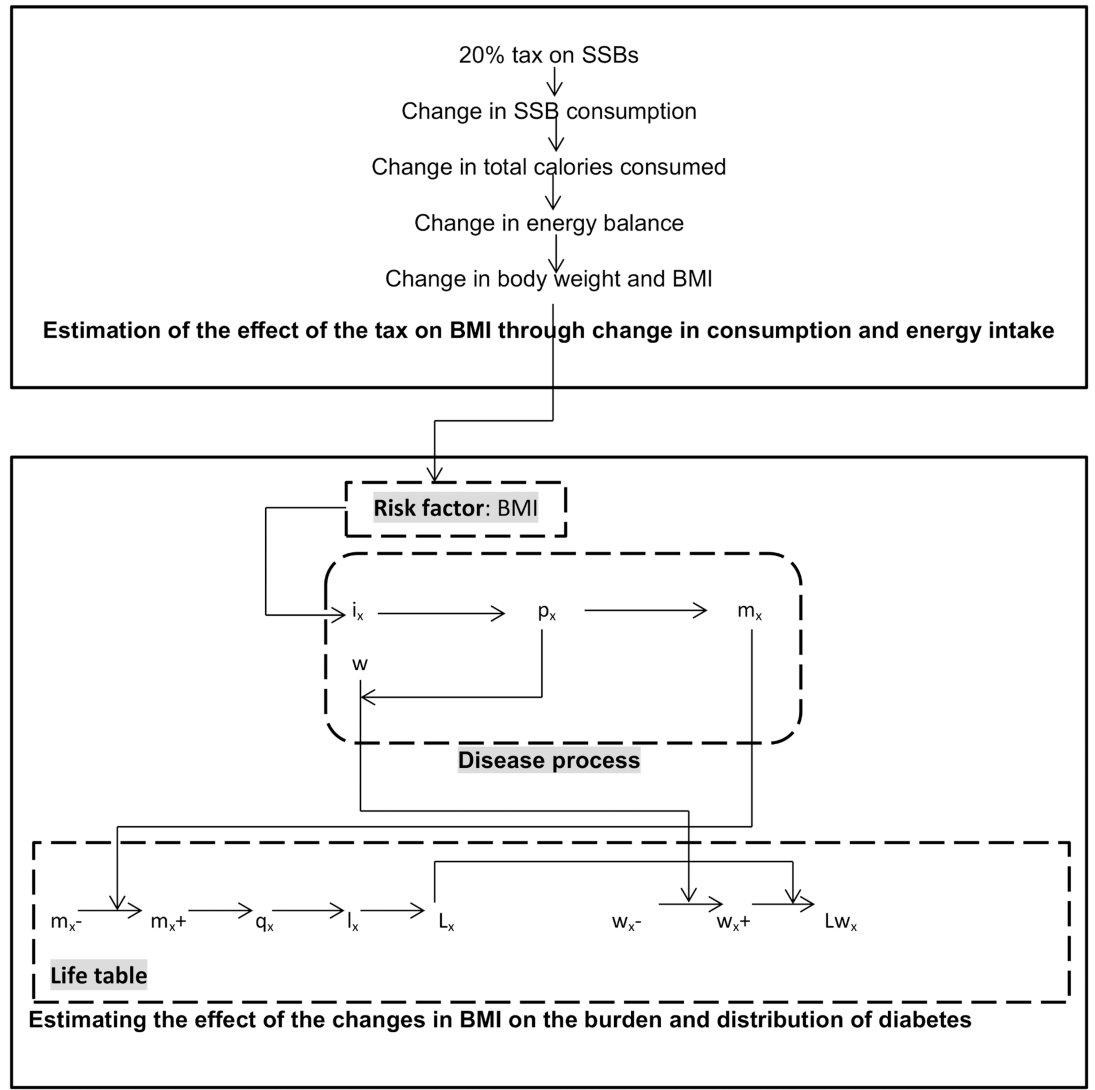
Schematic representation of the two- part modelling process. Fig1. shows the link between the SSB tax, changes in SSB consumption and BMI to the disease parameters and the life table. BMI is body mass index, x is age, i is incidence, p is prevalence, m is mortality rate, w is disability-adjustment, q is 1-year probability of dying, l is number of survivors, L is life years, and Lw is disability-adjusted life years. ‘-‘denotes a parameter without intervention and ‘+’ denotes a parameter with intervention.

A proportional multi-state life table-based Markov model was used to estimate the effect on T2DM of a reduction in body weight due to a 20% SSB tax [35]. Two populations are compared by the model: a reference population with the BMI distribution and disease pattern of the South African adult population aged 15 years and above in 2012, and an identical intervention population which receives the 20% tax. The modelling procedure was carried out in two steps: estimation of the effect of the tax on SSB consumption and energy intake and hence BMI, and the estimation of the effect of the BMI changes on the burden and distribution of T2DM. The model was implemented in Microsoft Excel (2010). Fig 1 is a schematic of the model showing the modelling steps for the intervention population. The reference population is modelled similarly except that no changes in BMI are incorporated. No trend in BMI is applied to either population.

### Intervention

We modelled the impact of a 20% SSB tax. Tax can be passed on in full to consumers, or manufacturers and retailers can absorb some of the tax by reducing profit margins. Our model assumed a 100% pass on rate to the consumers. Previous studies have modelled 10% and 20% tax rates with pass on rates ranging between 80–100% [24,26,36].

### Intervention effect on BMI

Previously published price elasticities were used to estimate changes in consumption of SSB, milk, diet drink and unsweetened fruit juice resulting from an increased SSB price [25,29]. Price elasticity measures the rate of response of product demand when price increases. Own-price elasticity is the change in demand that occurs for a product in response to price changes of the same product and cross-price elasticity is the change in purchases that occur for a product in response to price changes of another product. The price elasticity estimate values and standard deviations have been reported elsewhere and are shown in S1A Table [25].

Baseline consumption data of SSBs, milk and unsweetened fruit juice in adults aged 15 years and above were obtained from the 2012 SA National Health and Nutrition Examination Survey (SANHANES-1). The SANHANES-1, a baseline cross-sectional survey of the SANHANES, obtained questionnaire-based data through interviews in combination with anthropometric measurements. Data extraction procedures for the model have been previously described [29,37].

An SSB was defined as a non-alcoholic drink with added sugar and this comprised carbonated and non-carbonated sweetened drinks, sweetened fruit juices and squash concentrates based on the data available from the SANHANES-1. Change in SSB consumption was converted to change in energy intake using average energy density estimates for each drink category with the assumption that percentage change in energy intake equals the percentage change in volume consumed.

We calculated the energy density of SSBs, diet drinks and unsweetened fruit juice using the brand of Coca Cola products and assumed 1800 kilojoules (kJ)/litre for SSBs, 1340kJ/l for unsweetened juice and 4kJ/l for diet drinks. We used Coca-Cola because they account for approximately 60% of the off-trade soft drinks market in SA according to the SABMiller Quarterly Divisional Seminar Series, South Africa [38]. Furthermore, unpublished work done by Dr Celeste Naude based at the University of Stellenbosch found the mean energy density for an SSB to be 188kJ per 100ml. A sample of 90 carbonated drinks, sports drinks, concentrates, iced teas and sweetened fruit juices was used, using energy density values from the South African Medical Research Council Food Data System [39] and from SSB food labels. We assumed an energy density of 2540 kJ/l for whole milk based on Parmalat whole milk [40]. We assumed that all milk consumed was full cream milk based on The Report on South African Food Consumption Studies (1983–2000) which shows that more full cream milk is consumed per capita than skim milk [41]. The changes in energy intake for each beverage type were summed to give the net change in energy intake.

To estimate changes in BMI, we used previously published energy balance equations which state that a daily change in energy intake of 94kJ/day (SD 2.96) is associated with a change in body weight of 1kg in equilibrium for adults [42]. Data for baseline BMI distribution were obtained from the 2012 National Income Dynamics Study (NIDS) [43], SA’s first nationally representative panel study. We extracted and fit BMI data for adults aged 15 years and above to the log-normal distribution using procedures described elsewhere [29]. The log-normal distribution was compared to and chosen over the gamma distribution due to better fitting properties, also described in a previous study [29]. We used the NIDS Wave 1 and Wave 2 (2008 and 2010 respectively) data to estimate how the standard deviation changes as a function of the mean, described in S1 Text and the data presented in S2 Table.

### Effect of BMI changes on T2DM outcomes

Changes in BMI influence the incidence of T2DM, an effect which is quantified through the potential impact fraction (PIF). The PIF is the proportional change in disease risk due to change in exposure to a related risk factor [44]. We calculated the PIF based on the age and sex-specific changes in BMI distributions due to the 20% tax and the relative risks of T2DM, using the Epigear risk factor integral Excel add-in (http://www.epigear.com/index_files/risk_factor.html). The relative risks for T2DM due to increased BMI were obtained from the Global Burden of Disease 2010 study [45]. Because increased BMI does not only affect mortality through T2DM, we also included relative risks for BMI-related all-cause mortality in order to accurately estimate the life-years gained from reductions in DM-related mortality. The relative risk estimates were from the Prospective Studies Collaboration [46].

The PIF estimates then link to a disease model for T2DM which calculates the changes in disease incidence, prevalence and mortality resulting from the changes in BMI. Estimates of baseline T2DM incidence, prevalence and case fatality rate for SA are based on results previously published by Bertram et al [10]. They derived their estimates from DisMod II, a specialised software tool which generates an internally consistent set of epidemiological parameters for a condition using three parameters as inputs which parameters were prevalence, all-cause relative risk of mortality and remission in this case. Case fatality rate is defined as the risk of dying for persons with T2DM minus the risk of death for persons of the same age and sex without T2DM. This includes deaths due to diabetes related conditions such as stroke and heart disease.

The disease relative risk, all-cause mortality relative risk, incidence, prevalence and case fatality rate estimates used in the model are presented in S1B and S1C Table.

### Calculating disability-adjusted life years, life years gained and health care costs

The changes in disease-specific prevalence and mortality are incorporated into the life table for the intervention population. The life table summarises the changes in T2DM in DALYs, cost offsets and life years gained. The populations are divided into 5-year cohorts and each cohort is simulated until death or 100 years of age (lifetime).

Years of life lived are adjusted at each age for time spent in poor health due to disease or injury based on prevalent life years lived with disability (pYLD) derived from the 2010 Global Burden of Disease (GBD) data for SA [47]. Population estimates by age and sex for 2012 were obtained from Statistics South Africa [48]. An average disability weight for T2DM of 0.0215 was obtained from a previous study by Bertram et al [10]. All-cause mortality rates were obtained from the SA Medical Research Council (MRC).T2DM healthcare cost data representative of the general South African population were not available. A proxy measure was derived from T2DM health care data from the Council for Medical Schemes (CMS) showing the average amount in ZAR claimed for diabetes per person in 2012. The CMS compiles these data based on the International Classification of Diseases (ICD10) codes. These data were only stratified by age and not by gender or type of care claimed for. The CMS costs were assumed to be private sector costs based on the fact that over 90% of medical aid members use private sector facilities. Based on the assumptions that public sector costs are 70% of private sector costs and that 18% and 82% of the SA population use private and public sector facilities respectively [49], we calculated weighted average costs to use as a proxy for diabetes costs for the South African population. Both the health care costs and the DALYs were not discounted. The pYLD,T2DM healthcare costs and mortality estimates used in the model are given in S1D Table.

### Uncertainty and sensitivity analysis

Ninety-five percent uncertainty intervals were estimated using Monte Carlo simulation in the Ersatz programme (Barendregt JJ, Brisbane 2007), varying the own- and cross-price elasticity estimates [25], the conversion factor between energy consumption change and weight change [42], the consumption estimates by age and sex for all four beverages [37], and the relative risk estimates [45,46].

Deterministic sensitivity analysis was performed to assess the effect on the burden of T2DM of varying the tax rate, the unit volume of a serving of SSB due to there being several serving sizes in which SSBs are available, health care costs, the pYLD estimates, mean BMI trend estimates and the discounting rate. The BMI trend analysis was based on the trend observed between the NIDS Wave 1 version 5.0 and Wave 3 version 1.0 which were conducted four years apart. The trend was applied per year. S3 Table shows how the trend in BMI units was calculated.

### Ethics

This study was a secondary analysis of human participant data collected through two national surveys, the SANHANES-1 for baseline consumption data and the NIDS for baseline prevalence of obesity. Both sets of data were anonymised and de-identified before we used them for our analysis. The two national surveys themselves independently obtained ethics approval [37,50].

## Results

### Change in SSB consumption, energy intake and BMI

The daily average consumption of SSBs, unsweetened fruit juice and milk at baseline in South African adults was 184ml, 200ml and 204ml respectively. SSB consumption declines with increasing age, with the 15–24 age-group drinking the highest amount and those aged 65 and over drinking the least. Table 1, previously published [29], presents the estimated changes in energy intake and mean BMI resulting from the SSB tax.

**Table 1:** Estimated change in energy intake and body weight after 20% tax.

Age	Change in average energy intake	Change in BMI in kg/m2	
	in kJ/person/day	(95% uncertainty intervals)	
	(95% uncertainty intervals)		
	All	Male	Female
**15–19**	-32.04 (-43.26, -22.43)	-0.17 (-0.32, -0.04)	-0.19 (-0.35, -0.05)
**20–24**	-45.78 (-80.14, -12.04)	-0.17 (-0.32, -0.04)	-0.19 (-0.35, -0.05)
**25–29**	-43.28 (-75.71, -9.64)	-0.16 (-0.29, -0.03)	-0.18 (-0.32, -0.04)
**30–34**	-43.02 (-74.37, -9.55)	-0.16 (-0.28, -0.03)	-0.18 (-0.32, -0.04)
**35–39**	-35.48 (-67.73, -0.89)	-0.13 (-0.26, 0.00)	-0.15 (-0.29, 0.00)
**40–44**	-35.45 (-68.67, -1.80)	-0.13 (-0.26, -0.01)	-0.15 (-0.28, -0.01)
**45–49**	-33.04 (-63.36, -0.15)	-0.12 (-0.24, 0.00)	-0.14 (-0.27, 0.00)
**50–54**	-33.03 (-64.07, -0.85)	-0.12 (-0.24, 0.00)	-0.14 (-0.27, 0.00)
**55–59**	-27.73 (-57.71, 3.65)	-0.10 (-0.22, 0.01)	-0.12 (-0.25, 0.02)
**60–64**	-27.93 (-58.14, 4.07)	-0.10 (-0.22, 0.01)	-0.12 (-0.25, 0.02)
**65–69**	-15.98 (-45.74, 15.56)	-0.06 (-0.17, 0.06)	-0.07 (-0.20, 0.07)
**70–74**	-16.03 (-47.13, 16.83)	-0.06 (-0.18, 0.06)	-0.07 (-0.20, 0.07)
**75–79**	-16.23 (-46.99, 16.43)	-0.06 (-0.18, 0.06)	-0.07 (-0.20, 0.07)
**80+**	-15.78 (-45.16, 15.29)	-0.06 (-0.18, 0.06)	-0.07 (-0.19, 0.07)

Table adapted from: Manyema et. al (2014). The Potential Impact of a 20% Tax on Sugar-Sweetened Beverages on Obesity in South African Adults: A Mathematical Model. doi: 10.1371/journal.pone.0105287 [29]

The highest change in energy intake estimate was approximately 46kJ in those aged 20–24 years. From ages 65 and up the change is estimated to be less than 17kJ per day. Trends in BMI change closely follow those in energy intake with more significant changes occurring in the younger adults as well as in females.

### Reduction in incidence and prevalence of T2DM

**Fig 2:**
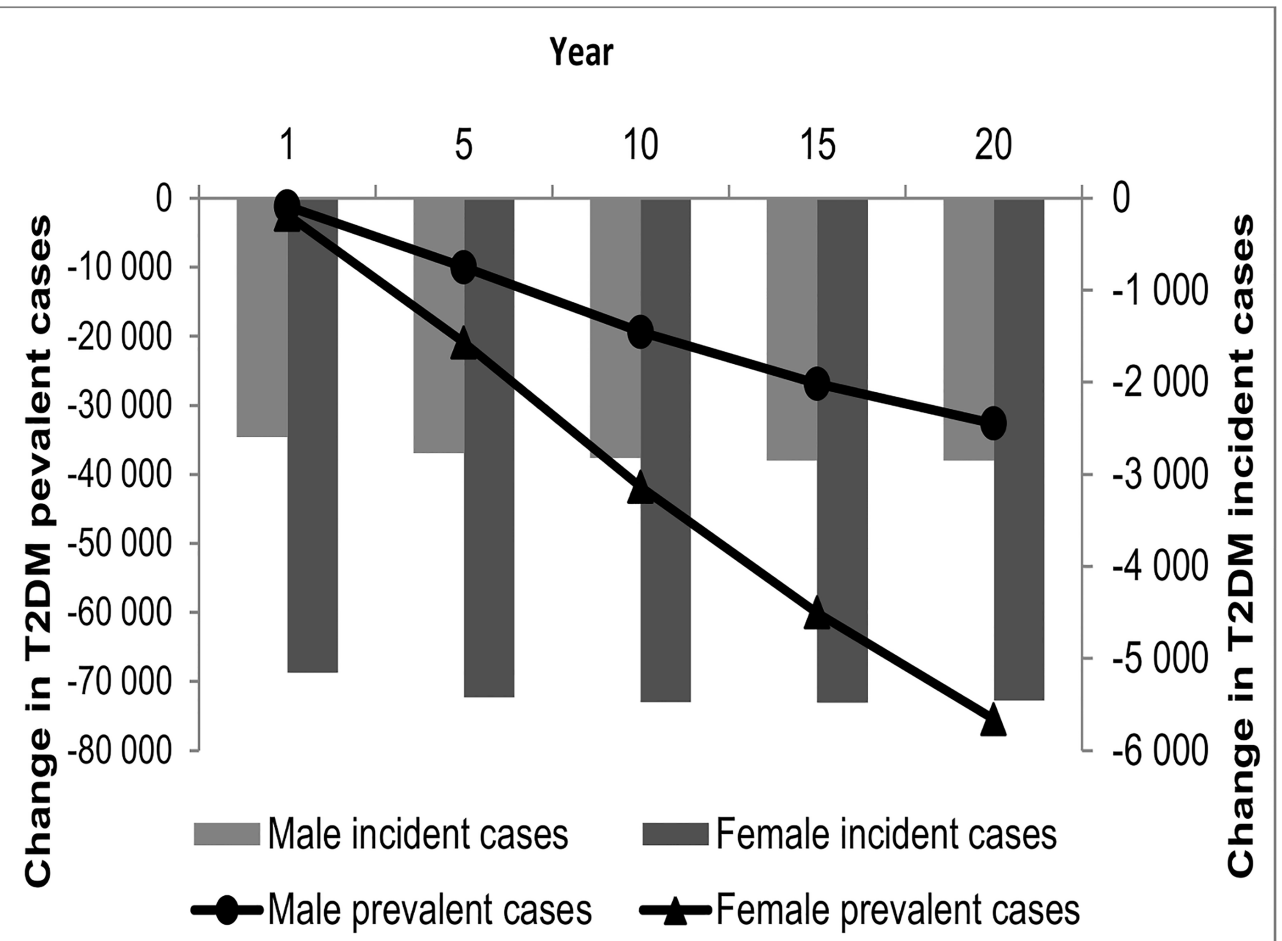
Change in the number of incident and prevalent T2DM cases over 20 years for males and females. Cumulative reduction in in prevalence is plotted on the left vertical axis and annual reduction in incidence on the right vertical axis. T2DM is type 2 diabetes mellitus.

Fig 2 shows the change in the numbers of incident (annual) and prevalent (cumulative) T2DM cases in South African men and women, over 20 years following the introduction of the tax, comparing the population exposed to the tax to the population not exposed.

We estimate that a 20% SSB tax would reduce the number of incident T2DM cases by about 106 000 in women (95% UI 70 000–142 000) and approximately 54 000 in men (95% UI: 33 000–80 000) cumulatively over 20 years. As Fig 2 shows, the number of incident cases averted year on year is relatively stable but the impact on prevalence rises and is significant. Twenty years after the introduction of the tax, the projected cumulative number of prevalent cases in all adults is nearly 108 000 lower compared to the scenario without the tax (95%: 71 000–144 000), a reduction in prevalence of 4.0% (95% UI: 2.7%-5.3%). The tax appears to have a much greater impact on diabetes in women than in men and this may primarily be because women have a higher mean BMI than men.

This is also reflected in the relative change in incidence which ranges between 6.7% and 6.1% in women between year 1 and year 20, and between 4.2% and 3.7% in men over the same period.

### Mortality, DALYs andT2DM healthcare costs averted

Table 2 presents the deaths (absolute numbers), DALYs andT2DM healthcare costs averted as a result of the 20% SSB tax.

**Table 2:** Annual T2DM mortality, DALYs and healthcare costs averted by the 20% tax over time.

Year	Mortality numbers male	Mortality numbers female	Healthcare costs male (million ZAR)	Healthcare costs female (million ZAR)	DALYs male	DALYs female
**1**	25 (15–38)	31 (20–44)	9 (5–14)	17 (11–24	406 (275–510)	704 (411–920)
**5**	226 (133–349)	292 (184–406)	83 (49–126)	163 (103–226)	3 481 (2 452–4 405)	6 458 (4 110–8 126)
**10**	455 (261–697)	611 (387–836)	166 (97–254)	343 (219–471)	6 676 (4 754–8 515)	13 034 (8 633–16 663)
**15**	649 (366–988)	903 (579–1 231)	238 (137–367)	514 (329–704)	9 112 (6 437–11 796)	18 335 (12 339–25 257)
**20**	803 (464–1 205)	1 156 (747–1 581)	299 (173–452)	672 (432–924)	10 785 (7 573–13 916)	22 172 (15 930–33 921)
**Life Time (cumulative)**	-	-	18 590 (10 337–28 265)	49 569 (31 391–67 830)	2.8 mil (1.9–4.0 mil)	4.0 mil (2.6–6.0 mil)

T2DM is type 2 diabetes, ZAR is South African rands, DALY is disability adjusted life-years, mil is million and lifetime is 100 years

We project that cumulatively, over 21 000 (95% UI: 14 000–29 000) adult T2DM deaths could be averted by the 20% tax over 20 years with the impact again being higher in women than in men and more deaths being averted with increasing time. Over the same period, an estimated total of 374 000 DALYs attributed to T2DM could be averted by the tax intervention (95% UI: 299 000–463 000), and over 7 million DALYs related to BMI increase in the adult lifetime (95% UI: 5.6 million-9.2 million).

The tax could also potentially save over ZAR10 billion in T2DM-related healthcare costs (95% UI: ZAR6.8–14.0 billion) equivalent to USD860 million (95% UI: USD570 million–USD1.2 billion) cumulatively over 20 years. The cumulative lifetime healthcare cost savings for women would be ZAR49.5 (USD 1.5) billion (95% UI: ZAR31 billion–R68 billion/ USD 2.6–5.7 billion) and over ZAR18 (USD 1.5) billion for men (95% UI: ZAR10 billion-ZAR28 billion/ USD 833 million -2.0 billion).

### Sensitivity analysis

Table 3 presents the results of the sensitivity analyses.

**Table 3:** Sensitivity analysis of various model parameters.

Parameter tested		Total T2DM incidence over 20 years	Total T2DM DALYs over 20 years	Total T2DM prevalence over 20 years	Total T2DM healthcare costs over 20 years (billion ZAR)
**Tax rate **					
	**10%**	-95 915	238 011	-64 694	-6.2
	**20%**	-160 106	374 751	-108 137	-10.3
	**30%**	-207 342	518 894	-140 177	-13.4
**Discount rate**					
	**0%**	-160 106	374 751	**-108 137**	-10.3
	**1%**	-160 106	374 751	-108 137	-9.3
	**2%**	-160 106	374 751	-108 137	-8.2
	**3%**	-160 106	374 751	-108 137	-7.2
**SSB portion size**					
	**200ml**	-42 938	117 167	-30 009	-2.8
	**250ml**	-88 024	221 297	-60 102	-5.7
	**330ml**	-160 106	374 751	-108 137	-10.3
	**500ml**	-309 493	768 966	-207 518	-20.0
**Health care costs**					
	**120%**	-160 106	374 751	-108 137	-12.6
	**110%**	-160 106	374 751	-108 137	-11.5
	**100%**	-160 106	374 751	-108 137	-10.3
	**90%**	-160 106	374 751	-108 137	-9.4
	**80%**	-160 106	374 751	-108 137	-8.4
**pYLD**					
	**100%**	-160 106	374 751	-108 137	-10.4
	**105%**	-161 077	402 359	-108 642	-10.4
	**110%**	-162 016	383 050	-109 330	-10.5
**BMI trend: BMI units/year**					
	**0**	-160 106	374 751	-108 137	-10.4
	**0.1**	-281 567	525 002	-280 733	-21.6
	**0.2**	-470 427	622 732	-548 691	-36.3
	**0.3**	-743 666	697 364	-944 074	-55.8

T2DM is type 2 diabetes mellitus, pYLD is prevalent years lived with disability, BMI is body mass index, SSB is sugar-sweetened beverage, DALY is disability adjusted life-years and ZAR is South African Rands.

Except for the tax rate and the BMI trend, the trends in the outcomes are more or less proportional to the size of the change in parameters, as would be expected for variations in the tested parameters. Increases in tax rate and BMI trend showed diminishing increases in the diabetes incident and prevalent cases, DALYs and costs averted. This reduced relative decrease with increasing trends can be attributed to the skewed shape of the population BMI distribution in combination with the exponential increase in the risk of disease with increasing BMI. Shifts in mean BMI due to same size changes in tax rate result in greater shifts in the upper tail of the distribution where the risk of disease is highest. Every additional increase in tax results in a smaller change in the upper tail of the BMI distribution, and impacts on a lower absolute risk of incident disease. Therefore even though incident and prevalent diabetes cases are still averted, there are diminishing returns. Conversely, the gains of the tax are expanded when set against a background of an upward trend in BMI.

## Discussion and Conclusion

The model projects that a 20% SSB tax would reduce the number of incident and prevalent T2DM cases by about 160 000 and 108 000 respectively over a 20 year period in South Africa. Because of decreased T2DM incidence, the prevalence rate would increase at a slower rate leading to a projected overall decrease in prevalence of 4.0% over that period. Our results are in line with those found in India where a 20% tax was projected to decrease T2DM prevalence by 1.6% over 10 years [28].

Over 21 000 adult deaths related to T2DM may potentially be averted, and nearly ZAR70 billion (over USD6 billion) worth of lifetimeT2DM healthcare costs. In a healthcare system that is already crippled by a significant communicable disease burden this cost saving would contribute substantially to reducing this burden.

Our model also predicts that over 7 million DALYs may be averted in the adult lifetime. The impact of the tax is greater on women than men because women have a higher population mean BMI compared to men.

### Study strengths and weaknesses

This study is the first in Africa to estimate the impact of fiscal policy on the burden of diabetes.

Our study has several strengths. We used nationally representative SA data to estimate baseline consumption of different drinks and baseline prevalence of obesity, which increases the generalizability of our results. The height and weight from the NIDS survey were measured, which should allow for accurate BMI estimates. The model accounts for substitution of SSBs with other drinks through the use of cross-price elasticities, which prevents the overestimation of the reduction in total energy consumption. The model demonstrates the effects of the tax by age and sex, and over time. We modelled the effect of the tax on T2DM over time to show the cumulative effect of the intervention.

A limitation of this study is the lack of SA-specific own- and cross-price elasticity data. The estimates used were pooled results derived from a systematic review and meta-analysis [25]. Our estimates were close to those used in previous work in Europe and the USA [24,26,51]. Variations in the price elasticities would have potentially impacted our results in several ways. A lower own-price elasticity, such as was found by Basu et al [28] would have led to smaller changes in SSB consumption and subsequently more modest changes in T2DM burden. Using higher price elasticities would have led to lower energy intake estimates than reported and hence greater reductions in T2DM.

We did not model the substitution effect to other sweetened drinks such as coffee, tea and hot chocolate. Similar studies have shown that the demand for tea and coffee goes up with SSB price increases [26,28]. This may have potentially attenuated the reduction in total energy intake from drinks. Price elasticities for skim milk were not available. Its inclusion in the model would have potentially diminished the effect of the tax.

Cross price elasticities may be culturally specific with some drink categories consumed in larger volumes than others in a particular society and this would potentially affect the shift in demand with increased price of SSBs. Our analyses from the SANHANES 2012 data showed that South African adults consume 88ml of milk, 200ml unsweetened juice and 184ml of SSBs per day on average. Data on consumption of other drink categories like coffee, tea, water were not available.

We did not account for different price elasticities between carbonated SSBs, drinks from concentrates, and sweetened fruit drinks. Briggs et al. found that an increase in the price of non-concentrated SSBs resulted in increased demand for concentrated SSBs and vice versa [24]. We also assumed the same price elasticities for both men and women, for all age and income groups and did not account for differences in baseline BMI. Some research suggests that demand for SSBs decreases the most in the lowest income group for all SSBs [26,28,52], while other studies found the effects similar across income strata, and still others found that demand decreased the most in the lower stratum for concentrated SSBs only [24].

Our model did not take into account the direct impact of sugar on diabetes that has been shown in other research [13,17,19], thus potentially underestimating the impact of the tax. Increase in BMI or weight gain is not the only cause of diabetes. We did not model other causes of diabetes and other diseases that are directly linked to diabetes such as cardiovascular disease, or diabetes complications like blindness and amputations. Again this is likely to have led to underestimation of the full impact of the tax. We estimate that the size of this underestimation is modest, however, as it would be dwarfed by the effects on life-years lost, and to a lesser extent also because the potential loss of quality of life is reduced by the effect of the disability weight for T2DM. With approximately 8 000 new cases of diabetes-related blindness each year [10] and a disability grant of about R12 120 per blind person per year [53], the SSB tax may potentially save an additional R97 million (USD 8 million) in T2DM-related costs every year.

We did not model the effect of the tax in children below the age of 15 who consume SSBs. Overweight or obese teens tend to become overweight or obese adults and the metabolic and physiologic changes associated with obesity in childhood and adolescence tend to track into adult life [54,55]. Prevention of obesity in children has the potential to go beyond its immediate effects to improve quality of life and prevent illness in later life [55].

We were not able to model trends in BMI due to unavailability of accurate data; however, we performed sensitivity analyses to see the effect on the results of variation in BMI. Analysis of the NIDS Wave 1 and Wave 3 surveys showed an increasing BMI trend of approximately 0.4 BMI units over four years. The implication of not modelling BMI trend is that we assume that BMI stopped changing in 2010 and T2DM incidence in the reference population remains constant. Guariguata et al projected a mean annual increase in diabetes prevalent cases of 55 000 [5]. The potential effect of these assumptions is to underestimate the potential impact of the tax in terms of reduction in T2DM incidence and prevalence. Our results show that without BMI trend, about 160 000 incident cases of diabetes may be averted over a 20 year period but over 280 000 may be averted when a BMI trend of 0. 1 BMI units per year is applied. We were not able to obtain empirical cost data for the model but we used estimates from medical schemes. These data were also tested in sensitivity analyses.

We modelled all diagnosed T2DM cases in South African adults. However, over 50% of cases are undiagnosed [7] and therefore our estimates of cost saving and healthy life gained may be low.

The cost data from the CMS were aggregated and we were unable to see differences in costs by gender. The costs averted in women may therefore have been underestimated as the results show that women would benefit most from the tax in terms of incidence and prevalence. We were also not able to see the type of care claimed for, whether or not sequelae were covered in the costs and which sequelae were included. It is possible that some complications and sequelae may have been missed by the coding and this would lead to underestimation of the costs.

### Public Health, Economic and Policy Implications

The majority of NCD deaths occur in LMICs and T2DM morbidity and mortality is highest in those aged 60 years and younger [4,7]. This burden of diabetes on the working force of emerging economies can have far-reaching consequences. A two-way relationship between health and economic development has been suggested [56,57]. Investment in health reduces the burden of disease and stimulates economic development, which further increases the ability to invest more in health. On the other hand, when an economy is too impoverished to invest in health, the disease burden may increase and poverty deepens [58,59]. The SSB tax has the potential to mitigate the rising diabetes epidemic in SA, reduce diabetes-related health care costs and contribute to economic growth.

About 50% of all diabetes cases in Sub-Saharan Africa were undiagnosed in 2013 and of those who are diagnosed, patients often present late with overt symptoms or complications [7]. Diabetes control in SA is equally poor with early screening and detection not available to everyone [60]. An SSB tax would have a population-wide impact on diabetes, helping to prevent the disease in those who are most at risk. Research has shown that diabetes prevention is best achieved through induction of weight loss [56] and a SSB tax has the potential of inducing population-wide weight loss.

Fiscal policy has been identified by the South African DOH as a cost effective ‘best buy’ that can be used to prevent obesity and NCDs [32]. Our study provides SA-specific evidence of how one such fiscal policy would impact T2DM. Tackling NCDs requires a multi-component approach, of which policy forms a crucial role in creating an enabling environment for consumers to make healthier choices. Tackling NCDs requires a multi-component approach, of which fiscal policy forms a crucial role in creating an enabling environment for consumers to make healthier choices. Other interventions which need to be implemented concurrently to have the greatest impact include food advertising regulations, food labelling, mass media campaigns and school-based interventions [32]. There are some challenges that will need to be overcome in order to ensure implementation of the SSB tax. These include, but are not limited to, vested interests, aggressive marketing, advertising of SSBs and corporate social responsibility (CSR) activities by industries. An increase in the price of SSBs will likely result in reduced demand for SSBs as shown by the price elasticities. This may in turn increase the demand for healthier alternatives and influence manufacturers to reformulate their products [61]. The SSB tax appears to be regressive [61]. However the poor are also disproportionately affected by NCDs, have less access to healthcare, less income available to seek quality healthcare and die prematurely from NCDs. A tax of this nature would potentially contribute to the reduction these health inequities.

The Sustainable Development Goals (SDGs) proposal, unlike the Millennium Development Goals, specifically mentions NCDs, with this target: “by 2030 reduce by one-third pre-mature mortality from NCDs through prevention and treatment, and promote mental health and wellbeing” [62]. The SA NCD Strategy also commits to a goal of reducing the relative premature mortality from NCDs by at least 25% by 2020. The SSB tax has the potential to contribute to these targets. Due to the impact of diabetes on economic productivity and health care costs, the intervention may also contribute to the goal to “promote sustained, inclusive and sustainable economic growth, full and productive employment and decent work for all” [62].

### Conclusion

Our results show that fiscal policy can have a significant impact on the achievement of the goals stated in South Africa’s NCD strategic plan. An SSB tax has the potential to help mitigate the T2DM burden in SA and contribute to the achievement of health and well-being for all. The South African government should seriously consider limiting SSB consumption through the implementation of such a policy, as this will have a direct impact on the obesity epidemic and subsequent reduction in associated non-communicable diseases.

## Supporting Information

S1 Table Estimates of the parameters used in the model. A. Price elasticities and energy intake conversion factor used in the model. B. T2DM and relative risk, incidence, prevalence and case fatality rate baseline estimates, where CI is confidence interval, T2DM is type 2 diabetes and BMI is body mass index. C. All-cause mortality relative risk associated with increased BMI used in the model D. All-cause pYLD, all-cause mortality rate and T2DM-related healthcare cost estimates used in the model, pYLD is prevalent years lived with disability, T2DM is type 2 diabetes and ZAR is South African Rands.
(DOCX)

S2 Table Calculation of the change in standard deviation in relation to change in mean BMI. Calculation of the rate of change of standard deviation as a function of mean BMI using lognormal fitted data of NIDS Wave 1 and 2, 2013 Release is presented, showing the data and formulas used.
(XLSX)

S3 Table Mean BMI trends in South African adults between 2008 and 2012 Based on the National Income Dynamics Study waves 1 and 3. Calculation of annual BMI trend between 2008 and 2012 to determine BMI trend to use in sensitivity analysis.
(XLSX)

S1 Text Estimation of changes in standard deviation of the mean as a function of the mean. This is a description of the procedure used to estimate the changes in the standard deviation as a function of mean BMI.
(DOCX)
