# Supplementary material for: Decreasing the Burden of Type 2 Diabetes in South Africa: The Impact of Taxing Sugar-Sweetened Beverages
Source: PLoS One. 2015 Nov 17;10(11):e0143050. doi: 10.1371/journal.pone.0143050 (PMC4648571; doi:10.1371/journal.pone.0143050)
Supplement: S1 Table — A. Price elasticities and energy intake conversion factor used in the model. B. T2DM and relative risk, incidence, prevalence and case fatality rate baseline estimates, where CI is confidence interval, T2DM is type 2 diabetes and BMI is body mass index. C. All-cause mortality relative risk associated with increased BMI used in the model D. All-cause pYLD, all-cause mortality rate and T2DM-related healthcare cost estimates used in the model, pYLD is prevalent years lived with disability, T2DM is type 2 diabetes and ZAR is South African Rands. (DOCX) [file pone.0143050.s001.docx]

S1 Table Estimates of the parameters used in the model

1. Price elasticities and energy intake conversion factor used in the model.

| Parameter | Mean Value(SD) |
| --- | --- |
| SSB own price elasticity | -1.30 (0.11) |
| Milk cross-price elasticity | 0.13 (0.1) |
| Fruit juice cross-price elasticity | 0.39 (0.19) |
| Diet drinks cross-price elasticity | -0.42 (0.10) |
| Daily energy intake required for 1 kg change in weight (kJ/kg/day) | 94 (2.96) |

1. **T2DM and relative risk, incidence, prevalence and case fatality rate baseline estimates**

| **Age** | **Relative risk per BMI-unit increase above 21(95% CI)**  **Male and female** | **Incidence Male** | **Incidence Female** | **Prevalence Male** | **Prevalence Female** | **Case fatality rate**  **Male** | **Case fatality rate**  **Female** |
| --- | --- | --- | --- | --- | --- | --- | --- |
| **0-4** | - | 0.0001 | 0.0002 | 0.0009 | 0.0021 | 0 | 0 |
| **5-9** | - | 0.0002 | 0.0003 | 0.0015 | 0.0034 | 0 | 0 |
| **10-14** | - | 0.0003 | 0.0006 | 0.0026 | 0.0056 | 0 | 0 |
| **15-19** | - | 0.0005 | 0.0009 | 0.0043 | 0.0092 | 0 | 0 |
| **20-24** | - | 0.0008 | 0.0014 | 0.0073 | 0.0148 | 0.0007 | 0.001 |
| **25-29** | 1.37 (1.28-1.46) | 0.0013 | 0.0023 | 0.0122 | 0.0236 | 0.0054 | 0.0055 |
| **30-34** | 1.34 (1.26-1.43) | 0.0023 | 0.0037 | 0.02 | 0.0366 | 0.0164 | 0.0142 |
| **35-39** | 1.32 (1.24-1.39) | 0.0037 | 0.0052 | 0.032 | 0.0545 | 0.0232 | 0.0169 |
| **40-44** | 1.29 (1.22-1.36) | 0.0055 | 0.0066 | 0.0492 | 0.0772 | 0.0253 | 0.0158 |
| **45-49** | 1.27 (1.21-1.33) | 0.0073 | 0.0076 | 0.0719 | 0.1033 | 0.026 | 0.015 |
| **50-54** | 1.24 (1.19-1.30) | 0.0089 | 0.0079 | 0.099 | 0.13 | 0.0259 | 0.0148 |
| **55-59** | 1.22 (1.17-1.27) | 0.0097 | 0.0076 | 0.1275 | 0.1542 | 0.0257 | 0.0153 |
| **60-64** | 1.20 (1.15-1.24) | 0.0099 | 0.0071 | 0.1539 | 0.1738 | 0.0272 | 0.0171 |
| **65-69** | 1.18 (1.14-1.22) | 0.0093 | 0.0064 | 0.1753 | 0.1883 | 0.0272 | 0.0181 |
| **70-74** | 1.15 (1.12-1.19) | 0.0089 | 0.0059 | 0.1912 | 0.1983 | 0.0293 | 0.0198 |
| **75-79** | 1.13 (1.10-1.16) | 0.0087 | 0.0058 | 0.202 | 0.2049 | 0.0325 | 0.0219 |
| **80+** | 1.10( 1.08-1.12) | 0.0092 | 0.0062 | 0.2089 | 0.209 | 0.0376 | 0.0258 |

CI is confidence interval, T2DM is type 2 diabetes and BMI is body mass index.

1. **All-cause mortality relative risk associated with increased BMI used in the model**

| **Age** | **Relative risk per BMI-unit increase for BMI 25-50 kg/m^2^**  **Male and female** | **95% LCI** | **95% HCI** |
| --- | --- | --- | --- |
| 35-59 | 1.07 | 1.06 | 1.09 |
| 60-69 | 1.06 | 1.05 | 1.08 |
| 70-79 | 1.06 | 1.05 | 1.07 |
| 80-89 | 1.03 | 1.01 | 1.05 |

1. **All-cause pYLD, all-cause mortality rate and T2DM-related healthcare cost estimates used in the model**

| **Age** | **pYLD Male** | **pYLD Female** | **All-cause mortality rate**  **Male** | **All-cause mortality rate**  **Female** | **Healthcare costs per prevalent case (ZAR)**  **Male and female** |
| --- | --- | --- | --- | --- | --- |
| **0** | - | - | 0.0427 | 0.0365 | 0 |
| **1-4/0-4** | 0.0671 | 0.0654 | 0.0044 | 0.0041 | 5 855 |
| **5-9** | 0.0678 | 0.0587 | 0.0011 | 0.0010 | 5 387 |
| **10-14** | 0.0778 | 0.0763 | 0.0011 | 0.0009 | 5 437 |
| **15-19** | 0.0824 | 0.0893 | 0.0020 | 0.0018 | 5 736 |
| **20-24** | 0.0962 | 0.1136 | 0.0043 | 0.0051 | 6 155 |
| **25-29** | 0.1327 | 0.1430 | 0.0085 | 0.0104 | 6 605 |
| **30-34** | 0.1520 | 0.1526 | 0.0142 | 0.0135 | 6 940 |
| **35-39** | 0.1739 | 0.1573 | 0.0183 | 0.0140 | 7 334 |
| **40-44** | 0.2026 | 0.1877 | 0.0200 | 0.0136 | 7 609 |
| **45-49** | 0.2027 | 0.1924 | 0.0222 | 0.0135 | 8 075 |
| **50-54** | 0.1966 | 0.1962 | 0.0255 | 0.0144 | 8 767 |
| **55-59** | 0.2162 | 0.2090 | 0.0318 | 0.0176 | 10 012 |
| **60-64** | 0.2170 | 0.1299 | 0.0387 | 0.0227 | 11 559 |
| **65-69** | 0.2305 | 0.2272 | 0.0521 | 0.0324 | 14 066 |
| **70-74** | 0.2194 | 0.2332 | 0.0611 | 0.0406 | 15 871 |
| **75-79** | 0.2126 | 0.2446 | 0.0878 | 0.0668 | 16 814 |
| **80-84** | 0.2043 | 0.2749 | 0.1310 | 0.0931 | 17 923 |
| **85+** | - | - | 0.2781 | 0.1962 | 17 498 |

pYLD is prevalent years lived with disability, T2DM is type 2 diabetes and ZAR is South African Rands
