# Supplementary material for: Decreasing the Burden of Type 2 Diabetes in South Africa: The Impact of Taxing Sugar-Sweetened Beverages
Source: PLoS One. 2015 Nov 17;10(11):e0143050. doi: 10.1371/journal.pone.0143050 (PMC4648571; doi:10.1371/journal.pone.0143050)
Supplement: S1 Text — This is a description of the procedure used to estimate the changes in the standard deviation as a function of mean BMI. (DOCX) [file pone.0143050.s004.docx]

**Text S1 Estimation of how the standard deviation of the mean changes as a function of the mean**

Using NIDS Wave 1 and Wave 2 data we estimated the relationship of the SD in relation to the mean. This relationship was applied to the data in the model to adjust the SD of the mean for both the reference population and the intervention populations as the mean changes over the 20 year period under investigation.

We calculated the mean BMI and SD for the two datasets by sex and 10-year age bands. For each age band we calculated the relative change in mean BMI and the SD for both male and female. The relative change of the SD as a function of the mean for each age band was then obtained using the following formula:

Relative change in SD

Relative change in mean

Using the sample size for each age band and the results obtained from the formula above we calculated a weighted average ratio of the change in SD in relation to the mean for males and females.

Table S2 is a table showing the data and formulas used in the procedure, in Excel format.
